# Interventions and recommendations to mitigate measles outbreak in Tanzania

**DOI:** 10.1016/j.amsu.2022.104964

**Published:** 2022-11-18

**Authors:** Jonaviva Anthony Thomas, Innocent Kitandu Paul, Alfred Jubilate, Zenno joseph Kavishe, Michael Kazinza, Shafiya Shahab Haider Abdi, Malneste James

**Affiliations:** Faculty of Medicine, Kilimanjaro Christian Medical University College(KCMUCo), Moshi, Tanzania; Faculty of Medicine, Catholic University of Health and Allied Science (CUHAS), Mwanza, Tanzania; Muhimbili University of Health and Allied Sciences, Dar es Salaam, Tanzania; Faculty of Medicine, Catholic University of Health and Allied Science (CUHAS), Mwanza, Tanzania; Faculty of Medicine, Catholic University of Health and Allied Science (CUHAS), Mwanza, Tanzania; Muhimbili University of Health and Allied Sciences, Dar es Salaam, Tanzania; Open University of Tanzania, Dar es salaam, Tanzania

Dear Editor,

Not long ago, the Ministry of Health of Tanzania called the attention of its citizens to be cautious of the outbreak of Measles disease in the country, the ministry reported 38 confirmed cases of measles across the country [1]. However, no death caused by the disease has been recorded yet in Tanzania. Measles, which is a highly contagious viral disease, has been an important cause of mortality and morbidity in several Sub-Saharan countries such as Ethiopia, Somalia, Nigeria and Democratic Republic of the Congo which notably borders Tanzania easterly [2,3].

The main causative agent of this disease is the measles virus that belongs in the genus *Morbillivirus,* found in the *Paramyxoviridae family*. Measles is significantly spread from person to person through respiratory droplets especially in a closed room, and when a patient is exposed near to safe individuals the chances of getting infected of the disease are high [[3], [4], [5], [6]]. A patient suffering from the disease usually displays the symptoms until 10–14 days after exposure. However, the symptoms range from coughing, runny nose and sneezing, headache and commonly Koplik spots, skin rashes, dry cough and diarrhea [4].

Cases of measles disease globally have spiked abruptly since the emergence of COVID-19 pandemic, due to shifting of the focus from other infectious diseases to the pandemic [3]. Tanzania did not experience such a tragedy like other Sub-Saharan countries after the crisis. However, that does not put it in the safe zone following active interaction between the countries, considering that the disease is transmitted from person to person. It is also not proven that the recent outbreak is an impact of the pandemic, which has not been a big challenge at all.

Tanzania's Ministry of Health, as part of its intervention to the situation, sent a team of experts in the most affected areas with high numbers of incidence rate, these particularly are Kigamboni, Temeke and Ilala with 8, 12 and 4 cases respectively. In addition to that, other regions which reported the cases were Bukoba, Handeni, Kilindi and Mkuranga which are all seen on Fig. 1 of Tanzania's map [1]. The teams aimed at improving the timeliness and effectiveness of investigation and response to the outbreak including detection and root cause analysis to identify the gaps to prevent future outbreaks.

**Fig. 1:**
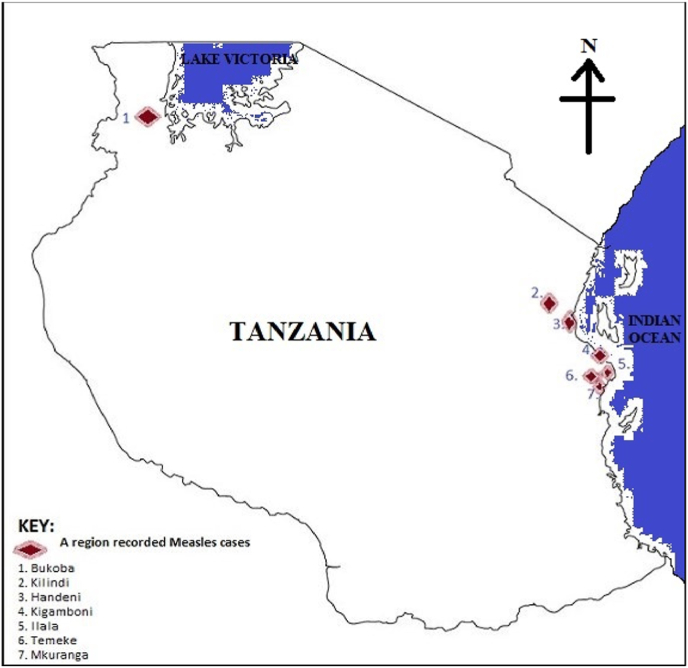
Showing a map of Tanzania with regions that recorded measles cases.

Furthermore, the ministry urged the Tanzanians to be cautious of the disease by avoiding close contact with the infected individuals. This was facilitated through TV broadcasting and Newspaper publications which disseminated the information addressed by the Minister herself. Most importantly, it also created awareness among the individuals on prevention of the disease [1].

Notably, the WHO has highly recommended the provision of routine mass vaccination of Measles-Containing Vaccine (MCV), both type 1 and 2, among the most vulnerable groups of people. The organization also set a coverage target of 95% of the population to be vaccinated against measles in the whole globe [7]. Unfortunately, Tanzania has not been able to achieve this target. One of the reasons could be because of the absence of regular attacks of the disease in the country.

Nonetheless, it is still recommended to ensure vaccination of the individuals especially the children under five years of age which are the ones at a greater risk of getting infected, pregnant women and unvaccinated mothers. Along with that, the ministry is required to create strategies that would lead to an increase in the mass vaccination coverage. Moreover, provision of vitamin A supplements to children is highly recommended as for the children lacking vitamin are prone to the infection [3,[8], [9], [10]].

Lastly, it is worthy to investigate the main igniting factors which led to the outbreak of measles in the country since it is clear that there is no definite statement declaring the main reasons for the emergence of the current outbreak. Understanding these factors would enable the health experts and their counterparts to mitigate the disease and create a safe environment. In addition to that, the Ministry of Health is also called to address to the public the risks of getting other outbreaks basically as an impact of the current outbreak. Meanwhile, should education on preventive health care be highly emphasized among the people, especially the pregnant mothers whose children are highly vulnerable to the disease, so as to be able to fight against other coming outbreaks. Notably, it was critically observed that among the regions affected mostly, the coastal regions have recorded a high number of cases thus bringing attention to both the citizens and the government to take action on the atypical observation to find out the reason behind cases in these regions.

## Ethical approval

Not applicable.

## Sources of funding

We did not receive any funding pertaining to the completion of this work.

## Author contribution

Conceptualization: All authors.

Writing-reviewing and editing:All authors.

Final approval of the manuscript: All authors have read and approved the final version of the manuscript.

## Conflicts of interest

No conflict of interest is to be declared within this submission.

## Registration of research studies

1. Name of the registry: Not applicable
2. Unique Identifying number or registration ID: Not applicable
3. Hyperlink to your specific registration (must be publicly accessible and will be checked): Not applicable

## Guarantor

Jonaviva Anthony Thomas.

Kilimanjaro Christian Medical University College.

Kilimanjaro, Tanzania.

Email; anthonyjonaviva@gmail.com.

## Consent

Not applicable.
